# Typification and taxonomic remarks on five species names in *Cytisus* (Fabaceae)

**DOI:** 10.3897/phytokeys.155.54224

**Published:** 2020-08-07

**Authors:** Gianniantonio Domina, Fabrizio Bartolucci, Patrik Mráz, Lorenzo Peruzzi, Fabio Conti, Otakar Šída, Gabriele Galasso

**Affiliations:** 1 Department of Agriculture, Food and Forest Sciences, University of Palermo, Viale delle Scienze ed. 4, I-90128 Palermo, Italy University of Palermo Palermo Italy; 2 Scuola di Bioscienze e Medicina Veterinaria, Università di Camerino - Centro Ricerche Floristiche dell’Appennino, Parco Nazionale del Gran Sasso e Monti della Laga, San Colombo, 67021 Barisciano (L’Aquila), Italy Università di Camerino Camerino Italy; 3 Herbarium collections and Department of Botany, Charles University Benátská 2, 128 01 Praha 2, Czech Republic Charles University Benátská Prague Czech Republic; 4 Dipartimento di Biologia, Unità di Botanica, Università di Pisa, Via Derna 1, 56126, Pisa, Italy Università di Pisa Pisa Italy; 5 Department of Botany, National Museum, Cirkusová 1740, 193 00 Praha 9, Czech Republic National Museum Prague Czech Republic; 6 Sezione di Botanica, Museo di Storia Naturale di Milano, Corso Venezia 55, 20121 Milano, Italy Museo di Storia Naturale di Milano Milano Italy

**Keywords:** *
Cytisus
*, *
Leguminosae
*, Mediterranean flora, nomenclature, Presl

## Abstract

This paper deals with the typification and taxonomy of five Mediterranean *Cytisus* species. *Cytisus affinis*, *C. candidus*, and *C. spinescens* nom. illeg., non Sieber ex Spreng. were described from Sicily by Karel Bořivoj Presl, *Cytisus spinescens* was described from Apulia (southern Italy) by Curt Polycarp Joachim Sprengel, and *C. villosus* was described from southern France by Pierre André Pourret (1788). Lectotypes are here designated for Presl and Sprengel’s names. A neotype is designated for *C. villosus*. The taxonomic revision of these five names confirmed that *C. villosus* Pourr. (= *Cytisus affinis* C.Presl) is the name to be used for the species occurring in the large part of the Mediterranean countries. *Cytisus spinescens* Sieber ex Spreng. (≡ *C. candidus* C.Presl = *C. spinescens* C.Presl, nom. illeg.) is the correct name for the amphi-adriatic species occurring in peninsular Italy, and along the NE coast of the Adriatic Sea. This species does not occur in Sicily and reference to this latter region in the protologues of both *C. spinescens* C.Presl and *C. candidus* C.Presl is a misinterpretation due, possibly, to exchange of labels.

## Introduction

The Italian vascular flora includes 17 native *Cytisus* L. species and subspecies (Bartolucci et al. 2018) belonging to seven sections (Cristofolini and Troia 2006), and *C. striatus* (Hill) Rothm., a naturalised alien in Liguria (Galasso et al. 2018). Half of these taxa are widespread in the Mediterranean region and occur in a large portion of Italy (e.g., *C. hirsutus* L. and *C. villosus* Pourr.); other taxa show a limited distribution and occur only in a few Italian regions (e.g., *C. pseudoprocumbens* Markgr.), or are narrow endemics (e.g., *Cytisus aeolicus* Guss. confined to the Aeolian Islands, Conte et al. 1998).

Several names in *Cytisus*, published during the 19^th^ and 20^th^ centuries, still lack a nomenclatural type and there are even doubts about the taxonomic position for some of these names (Peruzzi et al. 2015, 2019). Among them, there are three species described from Sicily by Karel Bořivoj Presl (1794–1852, standard botanical form C.Presl from Carl, Carel or Carolus) from Sicily, namely *C. affinis* C.Presl, *C. candidus* C.Presl, and *C. spinescens* C.Presl. These taxa were described only very briefly, in the form of footnotes within a list of taxa occurring in Sicily (Presl 1826: XIX). No locality was specified in the protologues. These names, as well as the related ones *C. spinescens* Sieber ex Spreng. and *C. villosus* Pourr., are typified here and their taxonomic relationship is discussed.

This contribution is part of the large project aimed at typifying all taxa described from Italy and recognising their *loci classici* in order to serve as a basis for further taxonomic studies (Domina et al. 2012; Passalacqua et al. 2014; Peruzzi et al. 2015, 2019; Brundu et al. 2017).

## Material and methods

We performed a survey of the original material in the herbaria PR (National Museum, Prague) and PRC (Charles University, Prague) (acronyms according to Thiers 2019+), hosting the Presl’s Sicilian collections (Stafleu and Cowan 1983). Further material has been searched in the main Italian and European herbaria that could host duplicates and/or the original material of *C. spinescens* Sieber ex Spreng. and *C. villosus* Pourr.: B, BM, BOLO, FI, G, K, MA, MAF-POURRET, NAP, P, PAD, PAL, RO, W, and WU. The articles of the *International Code of Nomenclature for algae*, *fungi*, *and plants* (herafter ICN) cited through the text follow Turland et al. (2018).

## Typification of the names *Cytisus affinis*, *C. candidus* and *C. spinescens* described by K. B. Presl, with a note on his gatherings

### Cytisus affinis

Taxon classificationPlantaeFabalesFabaceae

C.Presl, Fl. Sicul.: XIX. 1826. [October 1826]

3D593D16-2DA6-5713-9B35-42050A768163

= C. villosus Pourr., Hist. & Mém. Acad. Roy. Sci. Toulouse 3: 317. 1788.

#### Ind. Loc.

“[Sicilia]”.

#### Type

**Figure 1.**
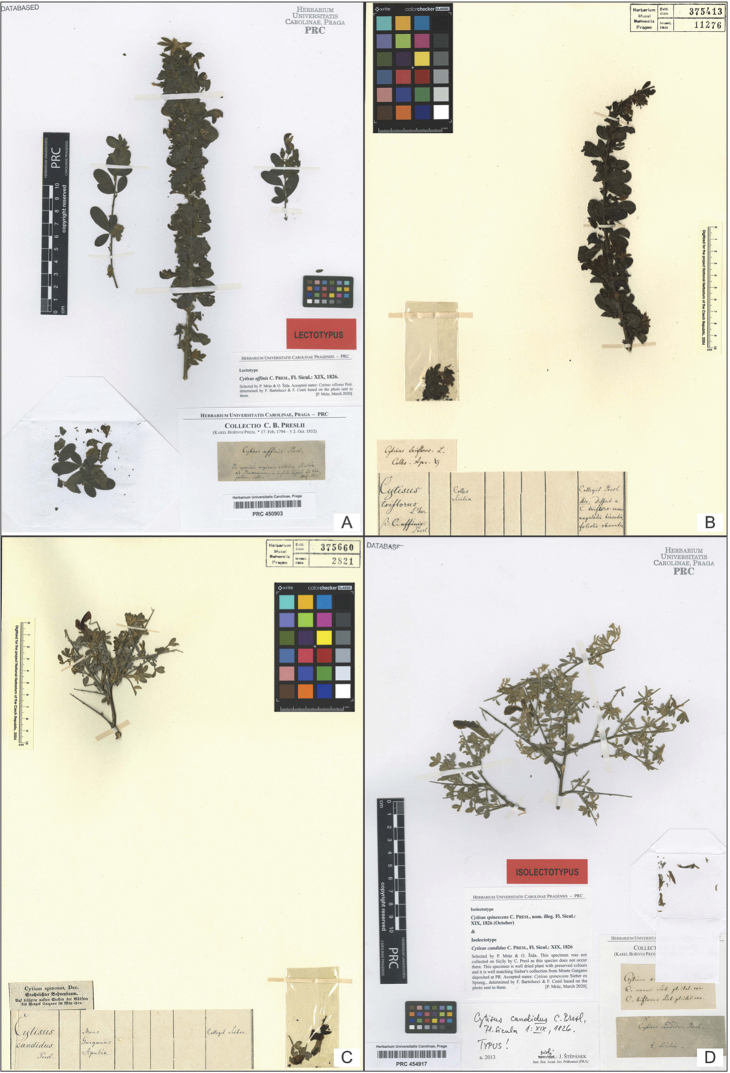
**A** The specimen of *Cytisus affinis* C.Presl (PRC 450903) here designated as lectotype of the name **B** The specimen of *C. affinis* C.Presl (PR 375413) **C** The specimen (PR 375660) here designated as lectotype of the names *C. candidus* C.Presl and *C. spinescens* Sieber ex Spreng. **D** The specimen of *C. candidus* C.Presl (PRC 454917) here designated as isolectotype of the name (all photos reproduced with permission).

(lectotype, here designated): Italy. [The label written by K.B. Presl] *Cytisus affinis* Presl. / In apricis regionis collinae Siciliae ad Panormum; in insula Capri ad Neapolim, etc., May 1817, *s.coll.* [*C. Presl*] *s.n.* (PRC 450903!, Fig. 1A); other original material PR 375413!) (Fig. 1B).

### Cytisus candidus

Taxon classificationPlantaeFabalesFabaceae

C.Presl, Fl. Sicul.: XIX. 1826. [October 1826]

C9E338B3-A532-5346-B6CE-430BE7B50A63

≡ [after typification, see below] C. spinescens Sieber ex Spreng., Syst. Veg., ed. 16 3: 225. 1826. [January–March 1826]

#### Ind. Loc.

: “[Sicilia]”.

#### Type

**Figure 2.**
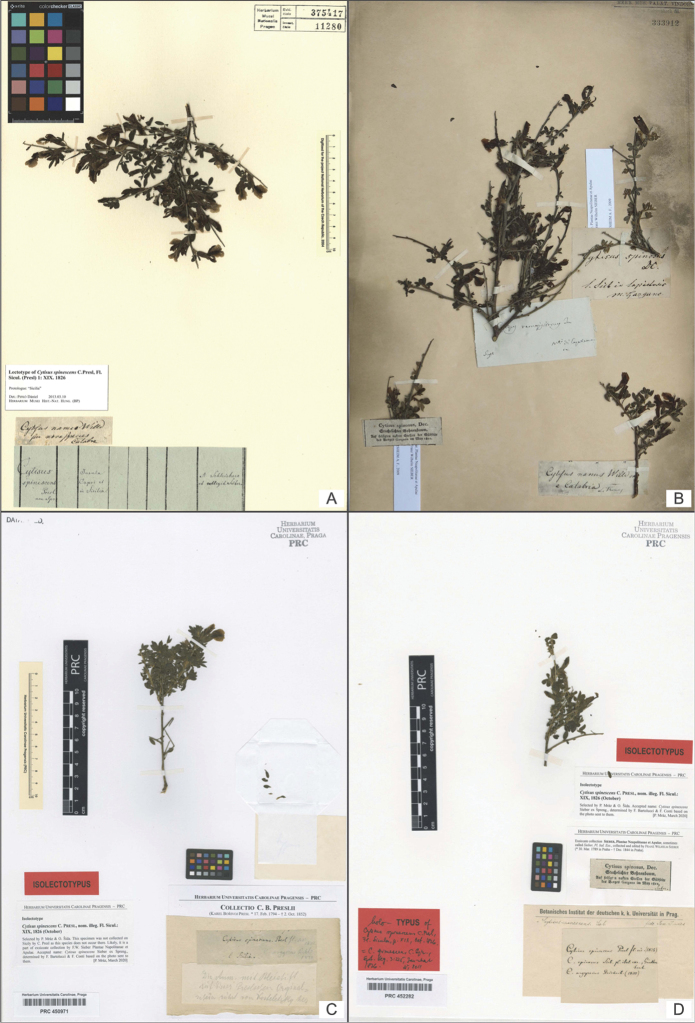
**A** The specimen PR 375417 here designated as lectotype of *Cytisus spinescens* C.Presl, *nom. illeg.***B** The herbarium sheet W 333912 bearing on the right bottom corner the isolectotype here designated of *C. spinescens* C.Presl and on the right top and on the left bottom corners the isolectotypes of *C. candidus* C.Presl **C** The specimen PRC 450971 here designated as isolectotype of *C. spinescens* C.Presl, *nom. illeg.***D** The specimen PRC 452282 here designated as isolectotype of *C. spinescens* C.Presl, *nom. illeg.* (all photos reproduced with permission).

(lectotype, here designated): Italy. [The label written by K.B. Presl] *Cytisus candidus* Presl. / Mons Garganus Apulia / collegit Sieber // [printed label of F.W. Sieber: PlantaeNeapolitanae et Apulae] *Cytisus spinosus*, Dec. Stachelicter Bohnenbaum. Auf felsigten nakten Stellen der Südseite des Berges Gargano, May 1812, *F.W. Sieber s.n.* (PR 375660!, Fig. 1C; isolectotypes PRC 454917! [Fig. 1D], JE 00021324 [digital photo!], W 333912 [digital photo!, the plant in the left bottom corner and the plant in the right top corner] [Fig. 2B]).

### Cytisus spinescens

Taxon classificationPlantaeFabalesFabaceae

C.Presl, Fl. Sicul.: XIX. 1826. [October 1826] nom. illeg. (Art. 53.1. of the ICN)

E4B17467-9873-5C11-BAE3-C2B4C62FBD2B

= Cytisus spinescens Sieber ex Spreng.
≡ Chamaecytisus spinescens Rothm., Feddes Repert. Spec. Nov. Regni Veg. 53(2): 143. 1944. [1 June 1944]

#### Ind. Loc.

: “[Sicilia]”.

#### Type

(lectotype, here designated): Italy. [The label written by K.B. Presl] *Cytisus spinescens* Presl non Spr. / Insula Capri et in Sicilia, a Schleichero et collegit Sieber. // [The label written by L. Thomas] *Cytisus nanus* Willd seu nova species / Calabre, s.d., *s.coll.* [*L. Thomas*] *s.n.* (PR 375417!, Fig. 2A; isolectotypes PRC 450971! [Fig. 2C], PRC 452282! [Fig. 2D], W 333912 [digital photo!, the plant in the right bottom corner] [Fig. 2B]).

#### Note.

During his professional life, K.B. Presl worked simultaneously as curator of Prague National Museum collections [at that time Patriotic Museum in Bohemia] (1823–1846) and in various positions in other Prague institutions (Maiwald 1904: 180; Skočdopolová 1995). At the beginning of his career, he taught economic botany in the garden of Count of Malabaila de Canal (from 1826), later at the Faculty of Medicine (from 1829) and Philosophy (from 1833). As noted by Skočdopolová (1995), Presl frequently transferred herbarium specimens from Museum collections to his office at the university because of more suitable conditions for his work. For this reason, K.B. Presl’s collections, including types, are variously distributed between today’s herbaria PR and PRC. After a detailed search for original material of the above mentioned names, we found seven specimens deposited in PR and PRC putatively belonging to different gatherings and identified as three distinct taxa. We found two specimens of *C. affinis* C.Presl (PRC 450903 [Fig. 1A], PR 375413 [Fig. 1B]) collected by the author in Sicily in 1817. The specimen deposited in PRC (Fig. 1A) bears a Presl’s label encompassing the species name and rather detailed locality, all written in italics, typical for his own collection (Domina and Štěpánek 2009). The specimen in PR (Fig. 1B) bears a label cut out from a specimen folder used at that time in C.M. Sternberg’s herbarium, including the species name (at varietal rank, “Cytisus triflorus L’her. β. C. affinis Presl.”), locality, collector and a short diagnosis against *C. triflorus* L’Hér. In addition, there is attached a small label from Presl’s exsiccata collection “Flora sicula”, suggesting that duplicates of this collection were distributed in the past and can be found elsewhere. Both specimens look very similar in respect of phenology and form of preparation and although they differ in the month of collection (May versus April), this likely originates from labelling of specimens in different times, and both specimens could be part of a single gathering. Both specimens are original material. They bear the name “*Cytisus affinis* Presl” written by himself, and in this case it seems unquestionable that the name *C. affinis* is based on specimens collected by Presl in Sicily. In any case, bearing two different dates, we prudentially consider them as two different gatherings. As the specimen in PRC [Fig. 1A] is more complete, we designate it as the lectotype of the name. From the morphological study of this specimen it is obvious that it agrees with the short original description, so that it can be stated that *C. affinis* C.Presl is a heterotypic synonym of *C. villosus* Pourr. Interestingly, in the PR label this taxon is subordinate to *Cytisus triflorus* L’Hér., and Presl himself later recognised *C. affinis* C.Presl as a synonym of *C. triflorus* [written without name’s authority], a species currently accepted under the name *C. villosus* Pourr. (see below), in his unpublished and undated second volume of *Flora Sicula*.

More problematic are the specimens belonging to the original material of *Cytisus candidus* and *C. spinescens*. We have found one specimen belonging to *Cytisus candidus* in PR (PR 375660!) [Fig. 1C] and one in PRC (PRC 454917!) [Fig. 1D], both showing well preserved colours. In PR and PRC, we have also found three specimens belonging to *C. spinescens*: (PR 375417 [Fig. 2A], PRC 450971 [Fig. 2C], and PRC 452282 [Fig. 2D] showing very brownish tint caused probably by very slow drying.

In addition, in W there is a sheet (W333912 photo!) [Fig. 2B] bearing four specimens with four labels bearing different names and collected in several localities of peninsular Italy: *Cytisus spinosus* DC. (two specimens from the Gargano), *C. ramosissimus* Ten. from the mountains near Castellammare, and *C. nanus* Willd. from Calabria. Although all specimens from PR and PRC bear Presl’s handwritten identifications, the plants belong to the same taxon and all agree with the protologues of both Presl’s *C. candidus* and *C. spinescens*. More specifically, both names were allegedly based on material originated from Sicily, as can be deduced from (i) descriptions of both taxa included in *Flora Sicula* (Presl 1826), and (ii) specification about the locality of *C. candidus* being collected in two carbonate promontories near Palermo (“Habitat in regione collina in saxosis apricis sterilibus ad promontorium Zafferana una vire, altera vire in monte Pellegrino”, see Presl, undated, unpub. msc. *Flora Sicula* vol. 2) or in Sicily in general (Presl’s annotations on two specimens deposited in PRC “E[x] Sicilia”; PRC 454917 and PRC 450971). Importantly, from the taxonomic point of view, both *Cytisus candidus* and *C. spinescens* C.Presl, are heterotypic synonyms of *C. spinescens* Sieber ex Spreng. (see below), a taxon which, besides Presl’s records from *Flora Sicula*, has never been reported from Sicily (Bartolucci et al. 2018). In fact, *C. spinescens* Sieber ex Spreng. is a taxon confined solely to the Italian peninsula (northwards to Latium, Umbria and Marche) and to the NE coast of the Adriatic Sea. In addition to the doubtful location (Sicily), it has become obvious from the elements specified below that these specimens were not collected by Presl himself, but by Franz Wilhelm Sieber (1789–1844) and by Charles-François-Louis-Alexandre [Luigi] Thomas (1784–1823) (cf. Burdet 1978; see also an annotation to the Table 1), respectively. We hypothesise that these discrepancies in locations and collectors have likely originated from dividing and postponing the labelling of these specimens by Presl himself. Such a mistake has previously been documented in *Asplenium lepidum* C.Presl, which was allegedly collected by him in Bohemia, but actually by Anton Rochel (1770–1847) in the region of Banat (currently in Romania and Serbia) (P. Mráz, unpublished data).

**Table 1.** Overview of elements involved in the nomenclatural history of four *Cytisus* taxa described by K.B. Presl and K.P.J. Sprengel from Italy and their taxonomic interpretation.

Barcode and nomenclatural type	Identification and morphology of specimen	Presl’s identification	Label(s)	Notes
PRC 450903 (Fig. 1A) **lectotype of *C. affinis* C.Presl**	*C. villosus* Pourr.	*C. affinis* C.Presl	“Cytisus affinis Presl. / In apricis regionis collinae Siciliae / ad Panormum, in insula Capri ad Nea- / polium, etc. Maj. 1817”	Standard Presl’s label from his own herbarium
PR 375413 (Fig. 1B) **other original material (syntype) of *C. affinis* C.Presl**	*C. villosus* Pourr.	*C. affinis* C.Presl	“Cytisus / triflorus L’Her. / β C. affinis / Presl. // Colles Siciliae // Collegit Presl. / Adn. Differt a C. trifloro ramis angulatis hirsutis foliolis obovatis”	Large Presl’s label cut out from the specimen folder used in Sternberg’s herbarium
			“Cytisus triflorus. L. / Colles. Apr.”	Presl’s label from his exsiccata collection *Plantae Siculae*, written in 1817 or early after
PR 375660 (Fig. 1C) **lectotype of *C. candidus* C.Presl, lectotype of *C. spinescens* Sieber ex Spreng.**	*C. spinescens* Sieber ex Spreng., well dried plants with preserved colours, well matching Sieber’s collection from Gargano	*C. candidus* C.Presl	“Cytisus / candidus / Presl. // Mons Garganus Apulia // Collegit Sieber”	Large Presl’s label cut out from the specimen folder used in Sternberg’s herbarium
			“Cytisus spinosus, Dec. / Stachelichter Bohnenbaum. / Auf felsigten Stellen de Südseite / des Berges Gargano im May 1812”	Sieber’s label from his exsiccata collection *PlantaeNeapolitanae et Apulae*, printed in 1812 or early after
PRC 454917 (Fig. 1D) **isolectotype of *C. candidus* C.Presl, isolectotype of *C. spinescens* Sieber ex Spreng.**	*C. spinescens* Sieber ex Spreng., well dried plants with preserved colours, well matching Sieber’s collection from Gargano	*C. candidus* C.Presl	“Cytisus candidus Presl fl. sic. / C. nanus Sieb. pl. ital. exs. / C. biflorus Sieb. pl. ital. exs.”	Presl’s handwritten label, which is very similar to the label on PRC 452282 (Fig. 2D) and was presumably written in 1832 or later. Reference to Sieber’s collection from Capri, also noted on specimen PR 375417 (Fig. 2A). Reference to *C. nanus* was probably wrongly ascribed to Sieber and, in fact, it refers to the specimen of L. Thomas
			“Cytisus candidus Presl. / E Sicilia.”	Presl’s handwritten label
PR 375417 (Fig. 2A) **lectotype of *C. spinescens* C.Presl, nom. illeg.**	*C. spinescens* Sieber ex Spreng., bleached and brownish plants, well matching Thomas’ collection from Calabria	*C. spinescens* C.Presl	“Cytisus / spinescens / Presl / non Spr. // Insula / Capri et / in Sicilia // A Schleichero / et collegit Sieber”	Large Presl’s label cut out from the specimen folder used in Sternberg’s herbarium written in 1826 or later. K.B. Presl referred to Schleicher, not to Thomas. J. C. Schleicher (1768–1834) was contemporary and also competitor of AbrahamThomas (1740–1824, father), Abraham Louis Emmanuel Thomas (1788–1859, son), Charles-François-Louis-Alexandre Thomas (1784–1823, son). Thomas’ family owned horticultural business in Bex, Switzerland (Moret, 1993, 1999), where was also active J.C. Schleicher. Gathering collected by one of Thomas was most probably sent to Prague by Schleicher (reference to Sprengel’s publication given)
			“Cytisus nanus Willd / seu nova species / Calabre”	Handwritten label probably by Ch.F.L.A. Thomas, but not entirely sure if written by him or by his brother A.L.E. Thomas. Based on the note on duplicate specimen kept in Wien (W 333912, Fig. 2B). According to Burdet (1978), the label is more probably written by A.L.E. Thomas, although presumably collected by Ch.F.L.A. Thomas, who worked in Calabria
PRC 450971 (Fig. 2C) **isolectotype of *C. spinescens* C.Presl, nom. illeg.**	*C. spinescens* Sieber ex Spreng., bleached and brownish plants, well matching Thomas’ collection from Calabria	*C. spinescens* C.Presl	“Cytisus spinescens. Presl / E Sicilia.”	Presl’s handwritten label; the annotations “fl. sic. 1825” and “C. argyreus Rchb. 1830” in pencil probably written by Kosteletzky were added later
PRC 452282 (Fig. 2D) **isolectotype of *C. spinescens* C.Presl, nom. illeg.**	*C. spinescens* Sieber ex Spreng., bleached and brownish plants, well matching Thomas’ collection from Calabria	*C. spinescens* C.Presl	“Cytisus spinescens Presl fl. sic. (1825) / C. spinosus Sieb. pl. ital. exs., Günther / herb. / C. argyreius Reichenb. (1830)”	Presl’s handwritten label from 1832 or later [reference to Reichenbach’s publication given]
			“Cytisus spinosus, Dec. / Stachelichter Bohnenbaum. / Auf felsigten Stellen de Südseite / des Berges Gargano im May 1812”	Sieber’s label from his exsiccate collection *PlantaeNeapolitanae et Apulae*, printed in 1812 or early after
PRC 455779	*C. spinescens* Sieber ex Spreng., glabrescent morphotype	–	“Cytisus biflorus. Tenore. / Zweiblüthiger Bohnenbaum / Auf der Insel Capri, den 6. April 1812.”	Sieber’s label from his exsiccate collection *PlantaeNeapolitanae et Apulae* and with Presl’s annotation ‘Sieber’, printed in 1812 or early after

In the case of *Cytisus candidus*, the specimen PR 375660 (Fig. 1C) bears, in addition to Presl’s label, also Sieber’s original label of “*Cytisus spinosus* DC.” from his exsiccata collection “PlantaeNeapolitanae et Apulae”. As stated on both labels, it was collected in Gargano, where this species (currently *C. spinescens* Sieber ex Spreng.) occurs (Fenaroli 1970; Bartolucci et al. 2018). Interestingly, Sieber’s original label is missing in the specimen found in PRC (PRC 454917, Fig. 1D), which bears two labels written by Presl only (Table 1). We found Sieber’s duplicates of this gathering also in JE (JE 00021324 Photo!) and W (W 333912 photo!, plant on the left bottom, Fig. 2B). Importantly, both these specimens bear Sieber’s exsiccata labels and the plants show the same colour and character as the specimens housed at PR (Fig. 1C) and PRC (Fig. 1D). We here selected the specimen at PR (PR 375660), bearing the original label from Sieber’s “PlantaeNeapolitanae et Apulae” collection, as the lectotype of *C. candidus* C.Presl. The specimen PRC 454917, as well as the duplicates in JE and W, are therefore isolectotypes of *C. candidus* C.Presl.

The three remaining specimens (PR 375417 [Fig. 2A], PRC 450971 [Fig. 2C], and PRC 452282 [Fig. 2D]) are again morphologically very homogeneous and were consistently identified by Presl as “*Cytisus spinescens* Presl”, although labelled as being collected from three different sites (see Table 1). Very important in this respect is the sheet W 333912 (Fig. 2B), with the specimen in the right bottom corner “*Cytisus nanus* Willd.” collected by Thomas in Calabria. Importantly, a similar label showing the same plant name and locality accompanies the specimen PR 375417 (Fig. 2A), whose plant shows similar / identical habitus as the one at W. The same can be argued for the specimens from PRC (PRC 450971 [Fig. 2C], and PRC 452282 [Fig. 2D]), albeit missing Thomas’ label. On the contrary, one of the PRC specimens (PRC 452282) bears Sieber’s label of his “PlantaeNeapolitanae et Apulae” collection (the same of *C. candidus* in PR 375660 [Fig. 1C] and W 333912 [Fig. 2B, plant on the left bottom]). Because this label is missing on *C. candidus* specimen from PRC (PRC 454917, Fig. 1D), we hypothesise that Sieber’s label attached to the specimen of *C. spinescens* C.Presl (PRC 452282, Fig. 2D) emerged from a mistake and was, in fact, exchanged with that of *C. candidus* (PRC 454917, Fig. 1D). Since the specimen PR 375417 [Fig. 2A] contains the best preserved plant and bears both Presl’s identification label and original label by Thomas, we designate it as the lectotype of the illegitimate name *C. spinescens* C.Presl. Consequently, we consider the specimens in PRC (Figs 2C, 2D) and W (Fig. 2B, the plant in the right bottom corner) as duplicates of Thomas’ collection from Calabria, and hence isolectotypes.

A possible scenario leading to the current “messy” state is as follows. During the work on his *Flora Sicula* (between 1817 and 1825–1826), K. B. Presl had access to five gatherings of *Cytisus* from Italy. Two of them (*C. affinis* C.Presl) were part of his own herbarium and were collected by him in Sicily, another two (one by Sieber, one by Thomas) were part of Prague National Museum collections and came from Italian mainland. The fifth is a Sieber’s gathering from Capri Island (Campania, southern Italy) and bears Presl’s annotation ‘Sieber’. It is deposited in PRC (PRC 455779) with no duplicate in PR. Our hypothesis is that Presl divided the museum specimens and transferred fragments to his own herbarium, and vice versa, donating duplicates of his own collection from Sicily to the Museum. We suppose also that during this “fragmentation” of specimens, he probably did not annotate carefully these fragments, and this may be the main reason for the chaotic situation concerning these collections.

Based on the morphology of the specimens of Presl’s *C. candidus* and *C. spinescens*, which agrees with the short original descriptions, we conclude that both names are synonyms of *C. spinescens* Sieber ex Spreng. Because *C. spinescens* C.Presl was described about seven months later than *C. spinescens* Sieber ex Spreng. (Stafleu and Cowan 1983, 1985), and because both names are based on different types (see also below), Presl’s name is a later and heterotypic homonym of *C. spinescens* Sieber ex Spreng., illegitimate according to Art. 53.1 of the ICN. Consequently, Presl’s name should not be used as an accepted name as it is currently treated in *The Plant List* (2019) or in the *International Legume Database* (Roskov et al. 2006) and in *Euro+Med Plantbase* (Euro+Med 2006). Concerning the name *C. spinescens* C.Presl, it is noteworthy that in his unpublished second volume of *Flora Sicula*, Presl wrote that its provenance was unclear for him (‘*locus specialis mihi amplius non constat*’) and unclear was for him also the status of *C. spinescens* Sieber ex Spreng. with respect to *C. candidus* (‘Quid vero *C. spinescens* Spreng. … est … An species sequens?’ [the next species in the manuscript is *C. candidus*]).

## Typification of the names *Cytisus spinescens* Sieber ex Spreng. and *C. villosus* Pourr.

### Cytisus spinescens

Taxon classificationPlantaeFabalesFabaceae

Sieber ex Spreng., Syst. Veg., ed. 16 3: 225. 1826. [January–March 1826]

786F91FA-28F7-5A11-B6A5-BC000F1AB011

≡ Spartium spinescens (Sieber ex Spreng.) Bertol., Fl. Ital. 7(3): 345. 1850. [June 1850]
≡ [after typification] Cytisus candidus C.Presl

#### Ind. Loc.

“Mons Garganus Apul.” Puglia.

#### Type

(lectotype, here designated): Italy. [The label written by K.B. Presl] *Cytisus candidus* Presl. / Mons Garganus Apulia / collegit Sieber // [printed label of F.W. Sieber: PlantaeNeapolitanae et Apulae] Cytisus spinosus, Dec. Stachelicter Bohnenbaum. Auf felsigten nakten Stellen der Südseite des Berges Gargano, May 1812, *F.W. Sieber. s.n.* (PR 375660!, Fig. 1C; isolectotype PRC 454917! [Fig. 1D], JE 00021324 [digital photo!], W 333912 [digital photo!, the plant in the left bottom corner and the plant in the right top corner][Fig. 2B]).

#### Note.

As Sprengel based his description on the exsiccata series collected and issued by F.W. Sieber, the best solution for typification would be to choose the specimen from Sieber’s collection seen by Sprengel himself. Unfortunately, after the death of his son, Sprengel’s rich herbarium was divided into many parts and sold in small portions to different specialists and institutions (Stafleu and Cowan 1985). The largest part, containing the collections of many botanists and among them also those by Sieber, was bought by B in 1890 (Urban 1891), and subsequently destroyed during World War II. We found unequivocal duplicates of this F.W.Sieber’s collection in herbaria PR, W and JE, and as shown above also in PRC, although incorrectly labelled later by Presl. It is interesting to note that Presl based his later homonym *C. spinescens* on a different gathering (Thomas’ collection), while he described *C. candidus* on a F.W.Sieber’s gathering. As the above designated lectotype of *C. candidus* belongs, without any doubt, also to the original material of *C. spinescens* Sieber ex Spreng, we designate it also as the lectotype of the latter name. *Cytisus candidus* C.Presl thus becomes a homotypic synonym of the prioritary name *C. spinescens* Sieber ex Spreng.

This brings also another nomenclatural consequence: when treating *C. spinescens* Sieber ex Spreng. as a member of the separate genus *Chamaecytisus* Link, the correct name is *Chamaecytisus spinescens* Rothm. This is because Rothmaler (1944) based his intended “new combination” on Presl’s illegitimate name, and thus accidentally published a replacement name (Art 58.1 of the ICN), which prevents making the combination based on legitimate Sprengel’s epithethon.

### Cytisus villosus

Taxon classificationPlantaeFabalesFabaceae

Pourr., Hist. & Mém. Acad. Roy. Sci. Toulouse 3: 317. 1788.

3CE5CA0F-7251-5287-9D00-BBCE1AE53847

≡ [after typification] Cytisus triflorus L’Hér., non Lam., nom. illeg.

#### Ind. Loc.

“Aux environs de Narbonne, à Fontlaurier”. France

#### Type

(neotype, here designated): Algeria. In montibus prope Algeriam, *s.d.*, R. L. Desfontaines, *s.n.* (G 00007761 [digital photo!] image: https://www.ville-ge.ch/musinfo/bd/cjb/chg/adetail.php?id=30955).

#### Note.

We did not find any original material for this name either in MAF (MAF-Pourret collection), and P (the general collection and the special Pourret’s collection named “*Chloris narbonensis*”), where Pierre André Pourret’s (1754–1818) collections are mainly kept (Stafleu and Cowan 1983), or in other relevant herbaria (BM, FI, MPU, and UPS; see Stafleu and Cowan 1983: 368). It seems, therefore, that the original material for this name is lost. This possibility is not surprising giving Pourret’s dramatic escape from France to Spain in 1789 and his forced exile (Galibert 1856), followed by several war events (Stafleu and Cowan 1983: 368). Because the original material of *C. villosus* is lost, we have decided to choose a neotype represented by the specimen G00007761 housed at G-DC. This specimen has been previously selected by Cristofolini and Fumeaux (Cristofolini and Troia 2006) as the lectotype of *C. triflorus* L’Hér. [1791]; an illegitimate name (a later homonym of *C. triflorus* Lam. [1786]) being conspecific with *C. villosus* Pourr. (see Polhill 1978; Cristofolini and Troia 2006). Importantly, as *Cytisus triflorus* L’Hér. has been accepted as the conserved type for the generic name *Cytisus* Desf., nom. cons. (Appendix III of the ICN), it becomes automatically a homotypic synonym of *Cytisus villosus* Pourr. – which is the accepted name of the generitype of this genus.

## Supplementary Material

XML Treatment for Cytisus affinis

XML Treatment for Cytisus candidus

XML Treatment for Cytisus spinescens

XML Treatment for Cytisus spinescens

XML Treatment for Cytisus villosus
